# Supplementary material for: PRDM16-DT is a novel lncRNA that regulates astrocyte function in Alzheimer’s disease
Source: Acta Neuropathol. 2024 Aug 29;148(1):32. doi: 10.1007/s00401-024-02787-x (PMC11362476; doi:10.1007/s00401-024-02787-x)
Supplement: Supplementary file 1 — Supplementary file1 (DOCX 1831 KB) [file 401_2024_2787_MOESM1_ESM.docx]

**Supplemental Figures**

**Supplemental Figure 1 (Fig. S1).**

**a.** Bar chart depicting the mean normalized expression of the lncRNA senriched in astrocytes as shown in Fig 1C. The data shows the expression in astrocytes. Note that lncRNAs *AL590617.2*, *VAC14-AS1* and *PRDM16-DT* show the highest expression levels when compared to the other lncRNAs. **b.** Violin plots showing the normalized expression of the lncRNAs depicted in panel A (except PRMD16-DT which is shown in Fig. 1D). Note the expression in astrocytes characterized by the maker gene AQP4.

**Supplemental Figure 2 (Fig. S2)**

**Fig. S2:** Bar plot showing *Prdm16os* expression in mouse astrocytes after treatment with tau fibrils compared to the corresponding vehicle control. (ns = not significant, unpaired t-Test). *Prdm16os* expression was normalized to *18S*. Error bars indicate SEM.

**Supplemental Figure 3 (Fig. S3)**

**
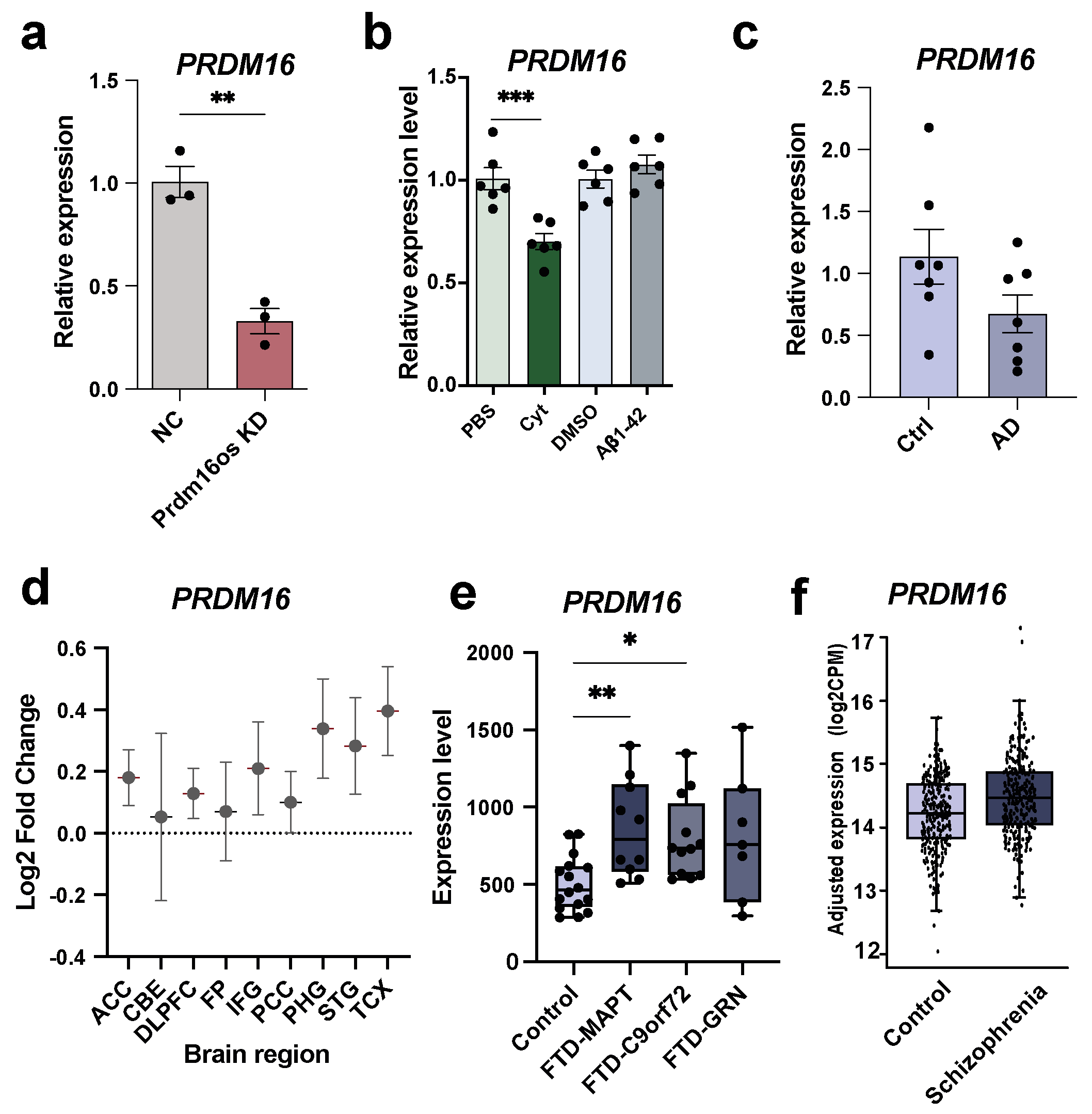
**

**Fig. S3: *Prdm16* expression in reactive astrocytes and disease. a.** Bar chart showing the expression of *Prdm16* after the KD of *Prdm16os* in primary astrocytes (**P<0.01, unpaired t-test). **b.** Bar plot showing *Prdm16* expression in mouse astrocytes after treatment with a 3 cytokine cocktail (Cyt) and Aß1-42 treatment compared to the corresponding vehicle controls. (***P < 0.0001, unpaired t-Test). **c.** Bar chart showing qPCR data on the expression of *PRDM16* in postmortem brain samples (prefrontal cortex, BA9) from control (n = 7) and AD patients (n = 7) (not significant, unpaired t-Test). **d.** Log2 Fold changes of *PRDM16* expression in different brain regions in AD patients compared to controls based on data from the Agora database (https://agora.adknowledgeportal.org/).(*P < 0.05). **e.** Bar chart showing the expression of *PRDM16* in postmortem tissue samples (frontal lobe) of FTD patients with MAPT (n = 10), C9ORF72 (n = 8) or GRN (n = 6) mutations compared to non-demented controls (NDC, n = 13). Data were obtained from the RiMOD database [34] [35]. **F.** Bar chart showing the expression of *PRDM16* in postmortem brain tissue of controls (n = 279) compared to schizophrenia patients (n = 258) obtained from a study by Wu et al. [36].

**Supplemental Figure 4 (Fig. S4)**


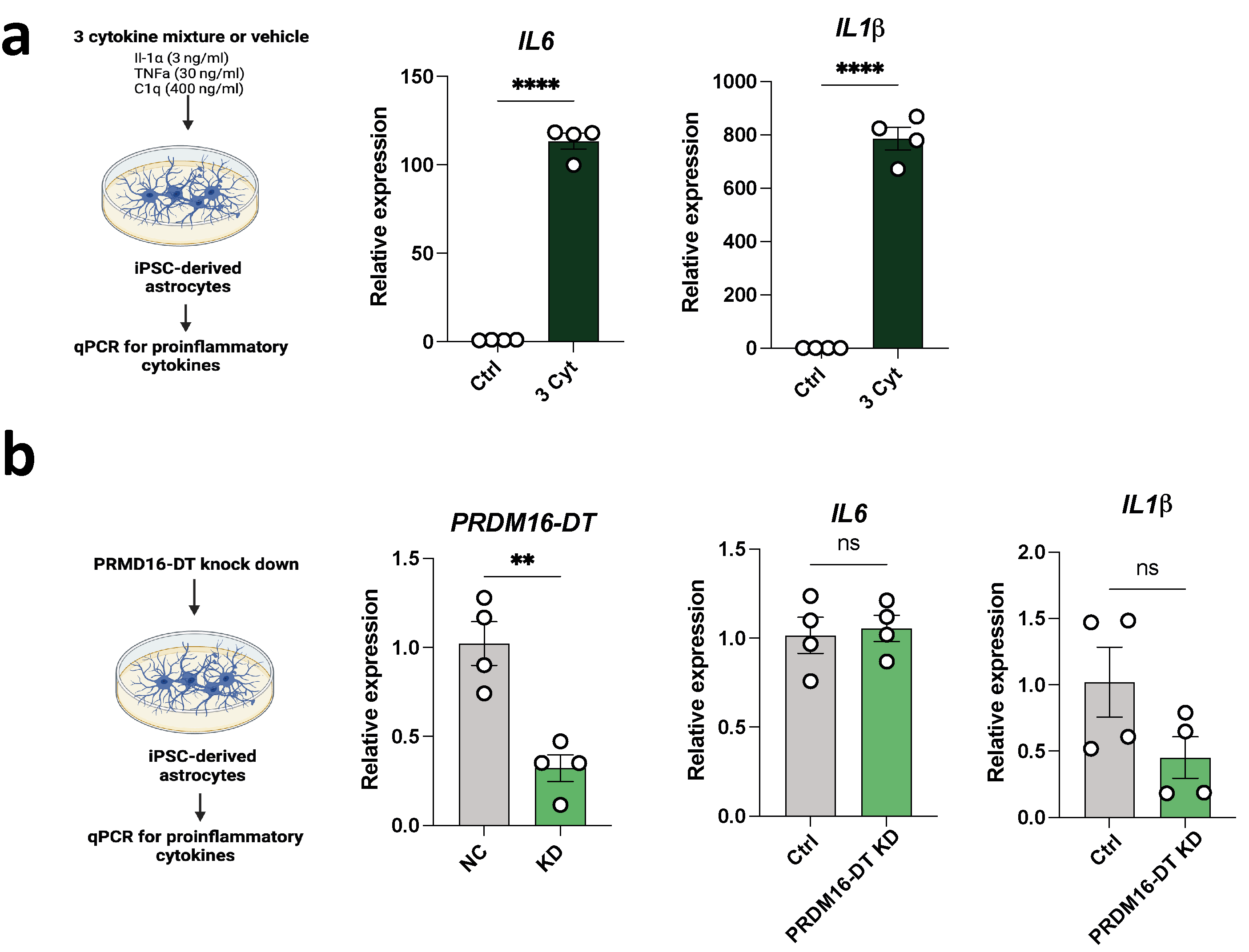


**Figure S4:** The knockdown of *PRDM16-DT* does not induce a pro-inflammatory phenotype in human iPSC-derived astrocytes. **a.** Scheme showing the experimental approach to stimulate human iPSC-derived astrocytes and the expression levels of pro-inflammatory cytokines after treatment with the 3 cytokine cocktail. **b.** Treatment scheme of PRDM16-DT KD in human iPSC-derived astrocytes followed by qPCR for IL6 and IL1b. KD: knockdown, NC: negative control. Statistical significance was assessed by a Student’s unpaired t test; **P < 0.01, ****P < 0.0001, ns = not significant. Gene expression was normalized to *18S* for *PRDM16-DT* or *GAPDH* for protein-coding genes.
